# Evaluating *Proteus mirabilis* phage vB_PmiA_PM1 efficacy against catheter-associated urinary tract infections in artificial urine and laboratory media

**DOI:** 10.1186/s12866-026-04843-w

**Published:** 2026-03-06

**Authors:** Salwa E. Gomaa, Christiana R. B. Youssef, Nader M. Sobhy, Sagar M. Goyal, Tieshan Teng, Fatma Al-zahraa A. Yehia

**Affiliations:** 1 Henan International Joint Laboratory for Nuclear Protein Regulation, School of Basic Medical Sciences, Henan University, Kaifeng, 475004 China; 2 Department of Microbiology and Immunology, Faculty of Pharmacy, Zagazig University, Zagazig, 44519 Egypt; 3 Department of Animal Medicine, Faculty of Veterinary Medicine, Zagazig University, Zagazig, Sharkia 44511 Egypt; 4 Veterinary Population Medicine Department and Veterinary Diagnostic Laboratory, College of Veterinary Medicine, University of Minnesota, St. Paul, MN 55108 USA

**Keywords:** *Proteus mirabilis*, Catheter-associated urinary tract infections, Crystalline biofilm, Phage therapy

## Abstract

*Proteus mirabilis* is a leading cause of catheter-associated urinary tract infections (CAUTI) because of its capability to develop extensive crystalline biofilms that obstruct catheters. Phages can effectively eradicate *P. mirabilis* biofilms in vitro; however, the clinical trial outcomes have been controversial. The current study aimed to investigate phage efficacy against *P. mirabilis* CAUTI in artificial urine medium (AUM) and laboratory media. A novel lytic *P. mirabilis* phage, vB_PmiA_PM1, was isolated and characterized. The phage had a narrow host range, capable of infecting only its main host strain, indicating high specificity. The phage also showed short latency, a large burst size, and physiochemical stability. The phage genome is linear double-stranded DNA (42,188 bp) with 59 open reading frames and closely resembles *Salmonella* phage vB_SpuP_Spp16. A putative depolymerase with a peptidoglycan hydrolase domain was identified within the phage tail protein, highlighting its role in the degradation of bacterial cells and the biofilm matrix. The bacteriolytic and biofilm-eradicating activities of this phage differed considerably between AUM and laboratory-rich media. Additionally, the growth dynamics of *P. mirabilis* in AUM exhibited less abundant growth as compared to that in trypticase soya broth. In addition, the formation of diverse struvite crystal morphotypes occurred as the pH increased. However, in the presence of vB_PmiA_PM1 phage, one morphotype of coffin-lid crystal was observed, which increased over time. Furthermore, phage activity on silicone Foley catheter sections demonstrated altered biofilm architecture and swarming migration in both media. These findings highlight the discrepancies between laboratory testing of phage and its clinical applications, which might fill the gaps in the future success of phage therapy against biofilm-associated CAUTI.

**Graphical Abstract:**

**Figure:**
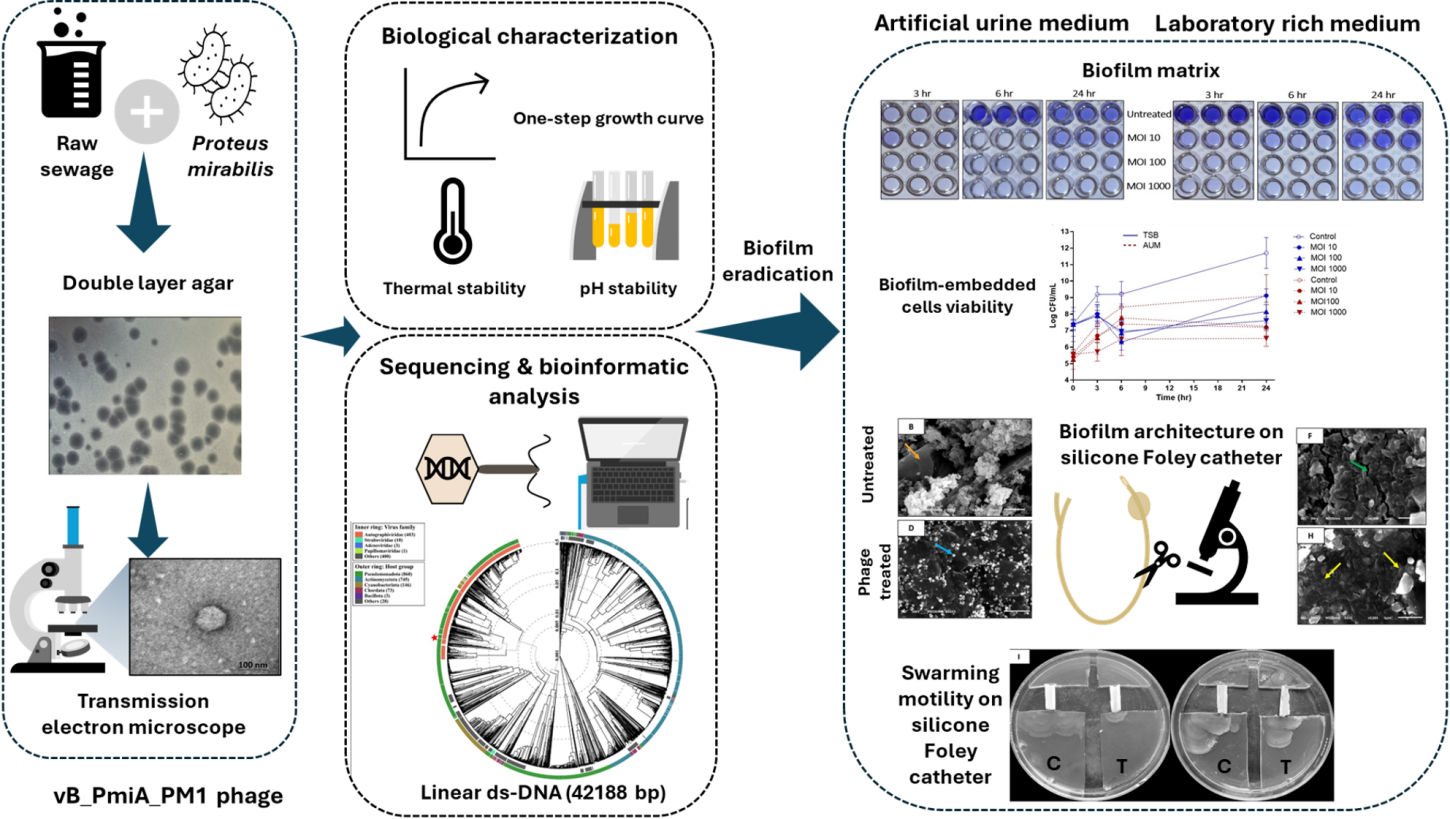
*Proteus mirabilis*-mediated CAUTIs demonstrate characteristic crystalline biofilm on the catheter surface. Phage therapy in CAUTIs has shown promising efficacy in the laboratory. However, clinical trials have not reflected these findings. Evaluation of phage activity under conditions that closely mimic the intricate environment of a catheterized urinary tract could pave the way for successful phage application in CAUTIs.

**Supplementary Information:**

The online version contains supplementary material available at 10.1186/s12866-026-04843-w.

## Introduction

Catheter-associated urinary tract infection (CAUTI) is the most prevalent nosocomial infection worldwide, with approximately 150–250 million cases annually [1]. The majority of microorganisms causing CAUTI originate from the patient’s intestinal microbiota colonizing the periurethral area or from the hands of health care providers. These organisms may gain entry into the patient’s urinary tract through the insertion or manipulation of indwelling urinary catheters [2]. Several risk factors associated with CAUTI have been identified; however, the duration of catheterization has emerged as the dominant risk factor. Nearly 25% of patients with short-term catheterization (< 7 days) develop bacteriuria, with infection risk increasing by 5% each additional day, reaching 100% in patients with long-term catheterization (≥ 30 days) [3]. Nevertheless, the risk of infection is not the sole complication for long-term catheterization; catheters are also prone to blockage owing to the formation of crystalline biofilm and encrustation. This often occurs in nearly half of long-term catheterized patients and primarily stems from infection with *Proteus mirabilis*, necessitating emergency referrals for catheter change [4, 5].

*P. mirabilis* is an opportunistic pathogen ubiquitous in nature, predominantly inhabiting the human gut [6, 7]. While generally commensal, *P. mirabilis* possesses a repertoire of virulence factors, most notably its distinctive swarming motility that facilitates urinary catheter colonization and adhesion to catheter or uroepithelial surfaces [8–10]. Another hallmark of *P. mirabilis* is its capacity to produce a potent urease enzyme in the presence of urine. This enzyme catalyzes urea hydrolysis, generating ammonia that elevates the local urinary pH. In the presence of increased pH, minerals that are normally soluble in urine are precipitated forming struvite and apatite crystals [11]. These crystals become embedded within the developing biofilm; their growth is enhanced and stabilized by the biofilm matrix [12, 13]. Ultimately, the biofilm progressively mineralizes, resulting in the development of extensive crystalline structures that obstruct catheters [11]. *P. mirabilis* can persist despite courses of antibiotics, multiple catheter replacements, and even periods without catheterization, underscoring the need for innovative therapeutic strategies such as phage therapy [14].

Phage therapy, the use of viruses that infect bacteria, has recently gained popularity in treating biofilm-related CAUTIs. Phages can infect and lyse bacteria present within biofilms. Beyond direct lysis, many phages encode polysaccharide depolymerases that are commonly present within phage as a structural protein in tail fibers, base plates, tail spikes, and tail tubular proteins [15]. These depolymerases exhibit various structures and catalytic mechanisms enabling them to degrade the capsular polysaccharides, extracellular polysaccharides, and/or lipopolysaccharides of Gram-negative bacteria. This enzymatic degradation exposes the bacterial cells, facilitating phage adsorption and attachment to bacterial receptors [15].

Phages have been shown to effectively eradicate *P. mirabilis* biofilms in vitro, as reported in several studies [16, 17]. Despite these encouraging findings, failure of phage therapy in clinical trials has also been reported [18]. We believe that the effectiveness of phage and/or enzymatic activity and bacterial behavior might be influenced by the urinary environment. Consequently, the choice of laboratory growth media or artificial urine media (AUM) could significantly affect the outcomes of phage research in vitro. Laboratory medium is nutrient-rich, providing an ideal environment for bacterial growth and phage activity. However, it might not accurately reflect the real urinary tract conditions or the intricacy of an actual infection. In contrast, AUM is chemically more representative of human urine, thus offering insights into real scenarios involving CAUTIs [19]. Additionally, previous studies reported diverse biofilm architectures and the number of swarmer cells in a standard laboratory medium compared to AUM [20].

In this context, understanding phage stability, activity, and host interactions in clinically relevant conditions simulating in vivo environments could bridge the knowledge gap between in vitro studies and clinical applications and help advance effective phage therapies for CAUTIs. The objective of this study was to isolate and characterize a novel lytic *P. mirabilis* phage, vB_PmiA_PM1, focusing on both its biological and genomic characteristics. Additionally, we investigated its in vitro bacteriolytic activity, the biofilm eradicating capability, and effectiveness against swarming migration using AUM that closely mimics human urine compared with standard laboratory medium such as Trypticase Soya Broth (TSB).

## Materials and methods

### Bacterial isolates

A total of 12 clinical *P. mirabilis* causing urinary tract infections were obtained from the Microbiology Department, Faculty of Pharmacy, Zagazig University. The isolates were re-identified to species level by MALDI-TOF MS. Bacterial isolates were routinely cultured in TSB. Trypticase soya agar (TSA) containing 1.5% or 0.3% agar was used in culturing bacteria and agar overlays, respectively. The antimicrobial susceptibility of the isolates was tested using the VITEK^®^ 2 system (Biomerieux, Marcy l’Etoile, France).

### Bacteriophage isolation and purification

Raw sewage samples were collected from a sewage pump station (Zagazig, Al-Sharkia, Egypt) and the bacteriophage was isolated according to a previously described protocol [21]. Briefly, wastewater samples were filtered through 0.22 μm-membrane filters (Millipore, Sigma, USA) and mixed with an equal volume of double-strength TSB supplemented with 1 mM magnesium sulfate and 1 mM calcium chloride. For phage enrichment, actively growing *P. mirabilis* cells were added to the processed wastewater and incubated overnight with continuous shaking. To assess phage lytic activity, the mixture was centrifuged, and the supernatant was filtered, before being spotted onto soft agar overlay containing the host strain.

Bacteriophages were purified using the double-layer technique as described by [22]. Three milliliters of molten soft agar mixed with 100 µL of exponential-phase *P. mirabilis* culture were overlaid on TSA plates. After solidification, the plates were incubated at 37 °C for 24 h and checked for the development of plaques on a confluent bacterial lawn. Individual plaques were isolated using sterile pipette tips, and the DLA technique was repeated until homogeneous phage plaques were obtained.

### High-titer phage stock preparation

The bacteriophage was propagated on the *P. mirabilis* host strain using the DLA technique to obtain a high-titer phage stock. Briefly, 100 µL of phage lysate was combined with 100 µL of exponentially growing host cells and mixed with pre-warmed molten agar, which was then overlaid on TSA plates. Following incubation and observation of confluent lysis, SM buffer was added to the plates and allowed to soak for 4 h. The SM buffer, along with the soft agar layer was harvested from the plates and centrifuged for 10 min at 4000 *xg*. The supernatant was sterilized by filtration, and the resulting phage stock solution was stored at 4 °C. Phage titer was expressed as plaque-forming units per milliliter (PFU/mL).

### Phage morphology examination

The morphology of the phage vB_PmiA_PM1 was assessed using a transmission electron microscope (TEM) as previously described [23]. Ten microliters of high-titer phage suspension (10^10^ PFU/mL) were applied to a copper grid and allowed to adsorb (5–10 min). Excess liquid was gently removed via filter paper and left to dry. For negative staining, a solution of 2% phospho-tungstic acid was applied. After 3–5 min, excess stain was removed and the grid was left to air-dry. The prepared grid was examined under a JEOL JEM 1010 electron microscope (JEOL Ltd, Japan) with a magnification of 6000x.

### Phage lytic spectrum determination

The lytic spectrum of vB_PmiA_PM1 phage was evaluated using the spot assay method. The phage was tested against clinical isolates of *P. mirabilis* and a diverse range of Gram-positive and Gram-negative bacteria obtained from the culture collection of the Microbiology Department, Zagazig University, Egypt. The formation of clear lytic zones on the bacterial lawn indicated lysis by the vB_PmiA_PM1 phage.

### Phage biological characterization

A one-step growth analysis was conducted to determine the phage latent period and burst size. Briefly, an exponentially growing *P. mirabilis* host strain (10^8^ CFU/mL) and vB_PmiA_PM1 phage at a multiplicity of infection (MOI) of 0.01 were incubated for 5 min before being centrifuged. The cell pellet was then resuspended in 10 mL of TSB and re-incubated. Aliquots were collected at 10 min intervals, and phage titers were assessed using the DLA technique [23]. Additionally, phage thermal stability and acid-base tolerance were evaluated via the DLA technique. The phage was exposed to a range of different temperatures (4–90 °C) and pH values (2–12) for 1 h. Different pH values were achieved using 1 M Hydrochloric Acid and 1 M Sodium Hydroxide and verified using pH meter. Following the one-hour stability testing, phage titer was monitored via DLA technique and expressed as PFU/mL.

### Genome sequencing and data analysis

The phage genomic DNA was extracted using a phage DNA isolation kit (Norgen Biotech, catalog No. 46800) according to the manufacturer’s instructions. Oxford Nanopore technology was used for sequencing using a rapid barcoding kit 96 (SQK-RBK110.96). The obtained sequence was assembled by CLC Workbench v. 1.1.2. The ORF finder tool was used to identify expected ORFs and annotate the genome (https://www.ncbi.nlm.nih.gov/orffinder/). Proteome-based phylogeny was used to compare the vB_PmiA_PM1 genome to ∼ 1937 phage sequences using VipTree (https://www.genome.jp/viptree/). Phylogenetic analysis based on the genome-to-genome distance calculator was performed by VICTOR analysis (https://ggdc.dsmz.de/victor.php*).* Phylogenetic analysis to locate genus and species was constructed using MEGA11 software [24]. Safety analysis was performed using the PhageLeads tool (https://phagecompass.ku.dk/*).* A comparative genomic map of phage vB_PmiA_PM1 protein and the closely related genome of *Salmonella* phage vB_SpuP_Spp16 was generated using Proteome Comparison tools available in the Bacterial and Viral Bioinformatics Resource Center (BV-BRC^3.46.3^; https://www.bv-brc.org/).

To predict phage depolymerase activity, Protein BLAST (BLASTP, https://blast.ncbi.nlm.nih.gov/Blast.cgi?PAGE=Proteins) identifying sequence similarities with known proteins and HHpred (https://toolkit.tuebingen.mpg.de/tools/hhpred) detecting putative structural and functional homologies were employed for phage tail proteins [25]. InterPro scan (https://www.ebi.ac.uk/interpro/) was used to annotate the functional domain [26]. Phyre2 (https://www.sbg.bio.ic.ac.uk/phyre2) was used to identify protein functional homology. Pairwise sequence alignment and secondary structure analysis were visualized using ESPript (https://espript.ibcp.fr/) to depict conserved residues, and sequence motifs to facilitate the identification of functionally important regions, and potential depolymerase domain.

The 3D structure of the protein was predicted via AlphaFold [27]. Protein physiochemical properties, including molecular weight, isoelectric point, and hydrophobicity were predicted using ProtParam on Expasy bioinformatics software tools [28]. Transmembrane topology and signal peptide predictions were performed to provide insight into the potential protein localization and functional roles in phage infection and depolymerase activity using DeepTMHMM [29].

### AUM assessment

Two different formulations of AUM were used to mimic the in vivo conditions of CAUTIs. The first AUM medium was devised by Griffith *et al*. and contained (g/L): calcium chloride 0.49, magnesium chloride hexahydrate 0.65, sodium chloride 4.6, disodium sulfate 2.3, trisodium citrate dihydrate 0.65, disodium oxalate 0.02, potassium dihydrogen phosphate 2.8, potassium chloride 1.6, ammonium chloride 1.0, urea 25, and gelatin 5.0. The pH was adjusted to 6.1 and sterilized by membrane filtration. TSB was prepared separately, autoclaved, and added to the medium to a final concentration of 1.0 g/L [30]. The second AUM, as described by Sarigul *et al.*, contained (g/L): sodium sulfate 1.70, uric acid 0.25, trisodium citrate dihydrate 0.72, creatinine 0.88, urea 15.0, potassium chloride 2.31, sodium chloride 1.76, calcium chloride 0.19, ammonium chloride 1.27, potassium oxalate monohydrate 0.04, magnesium sulfate heptahydrate 1.08, sodium dihydrogen phosphate dihydrate 2.91, and disodium hydrogen phosphate dihydrate 0.83 [31]. Phage stability and bacterial survival were assessed using different AUM formulations for optimization purposes. Viable count and DLA techniques were conducted to determine host strain viability and vB_PmiA_PM1 phage stability, respectively, over a 24-hour period. The AUM formulation that fulfilled the expected optimal host strain growth and phage stability was selected for subsequent experiments.

### Phage therapeutic efficacy assessment

#### Time-killing assay

Because of the intricate environment existing in the urinary tract, the interplay between phages and their hosts is more sophisticated and complex. Phage-host interaction was evaluated in the AUM formulation, described by [30], and in TSB via time-killing assay. Overnight host bacterial culture was mixed with vB_PmiA_PM1 phage at different MOIs (10, 100, and 1000). Bacterial viability was monitored in both media after 0, 3, 6, and 24 h and compared to control groups.

#### pH and crystal formation assessment

In the context of CAUTIs, *P. mirabilis* exhibits a characteristic urease enzyme, which induces alkalinization of urine, followed subsequently by crystallization of urine components. The effect of vB_PmiA_PM1 phage on pH changes and crystal formation caused by the host strain was assessed [32]. The pH changes in AUM media during phage-host interaction were determined using a pH meter and compared to the untreated groups. To monitor crystal formation, AUM was mixed with the host strain and vB_PmiA_PM1 phage at an MOI of 1000 and added into a conical tube without stirring. After 3, 6, and 24 h of incubation, crystal morphology was observed in AUM via light microscopy.

#### Mature biofilm eradication assay

The capability of the vB_PmiA_PM1 phage to eradicate mature biofilm in AUM and TSB was investigated. Free phages, biofilm-embedded cells, and biofilm mass were monitored in both media at specific time intervals (0, 3, 6, and 24 h) as described in [33]. The overnight culture of the host strain was added to the microtiter plate. After incubation, planktonic cells were aseptically removed, and wells were washed twice with sterile phosphate-buffered saline (PBS). The vB_PmiA_PM1 phage was added to the mature biofilm at different MOIs (10, 100, and 1000). At the specific time intervals, media were collected and centrifuged. The bacterial cell pellet was discarded, and supernatants containing free phages were subjected to phage titration. To quantify biofilm-embedded cells, the wells were washed with PBS and scraped to free the attached cells. Viable bacterial cells were suspended in PBS, serially diluted, and spread on TSA plates for counting. Additionally, total biofilm mass was assessed by crystal violet staining. The stained biofilm was measured spectrophotometrically at an optical density of 570 nm. Positive control groups, with no phage added, for the host strain in AUM and TSB were included.

#### Phage activity on silicone Foley catheter biofilm

Phage vB_PmiA_PM1 activity on silicone Foley catheter (SFC) biofilm was examined against biofilm architecture and swarming migration as described by [34] with slight modifications. SFC was cut into sectors, each 1 cm in length, and immersed in host-inoculated AUM and TSB. Following incubation and biofilm formation, SFC sections were aseptically removed, washed with PBS, and treated with phage vB_PmiA_PM1 (MOI 1000) for 6 h. SFC sectors were washed and divided into two sets. The first set was dehydrated and fixed as described by [34]. The biofilm architecture was observed using scanning electron microscopy (SEM). The second set was placed in sectioned TSA plates, where each sector served as a bridge. Five microliters of sterile medium were added into the catheter lumen and assessed for swarmer cell migration after proper incubation.

### Statistical analysis

Statistical analysis was performed using GraphPad Prism 8 software using Student ’s *t*-tests or one-way ANOVA. Data was presented as the mean ± standard errors of three independent experiments. *P* < 0.05 was considered significant.

## Results

### Identification and antimicrobial susceptibility of *P. mirabilis* clinical isolates

**Fig. 1:**
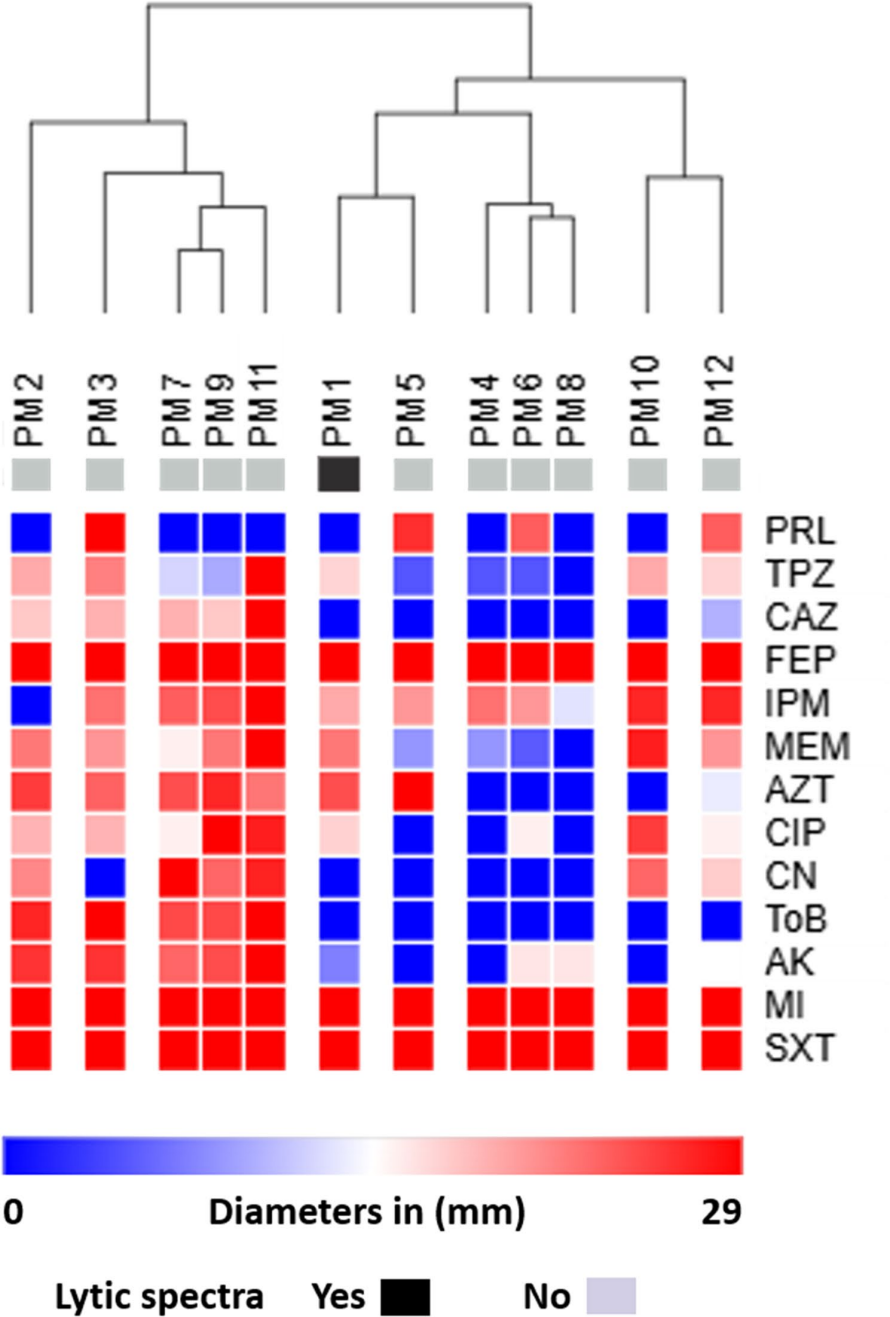
Heat map with hierarchical clustering based on antimicrobial susceptibility patterns of clinical *P. mirabilis* isolates. The color gradient below the diagram represents the diameters of inhibition zones (mm), with blue (lower values) and red (higher values). Lytic activity was annotated by color strips. The heat map was generated by Morpheus using hierarchical clustering with the Euclidean distance metric. PRL, Piperacillin; TPZ, Piperacillin-Tazobactam; CAZ, Ceftazidime; FEP, Cefepime; IPM, Imipenem; MEM, Meropenem; AZT, Aztreonam; CIP, Ciprofloxacin; CN, Gentamicin; TOB, Tobramycin; AK, Amikacin; MI, Minocycline; SXT, Trimethoprim-Sulfamethoxazole; R, Resistant; I, Intermediate; S, Sensitive

Twelve clinical *P. mirabilis* isolates associated with urinary tract infections were reconfirmed via MALDI-TOF MS to the species level as *P. mirabilis*. The antimicrobial susceptibility testing revealed significant resistance to various antibiotics, including piperacillin, ciprofloxacin, and sulfamethoxazole-trimethoprim (100% each), followed by ceftazidime, cefepime, and minocycline (91.7% each), gentamycin (83.3%), tobramycin (75%), and amikacin (58.3%). Furthermore, 41.7% of the tested isolates were resistant to both imipenem and aztreonam, while 33.3% were resistant to meropenem. Additionally, piperacillin-tazobactam resistance was detected in less than 9% of the isolates (Fig. 1).

### Isolation and morphological characterization of vB_PmiA_PM1

**Fig. 2:**
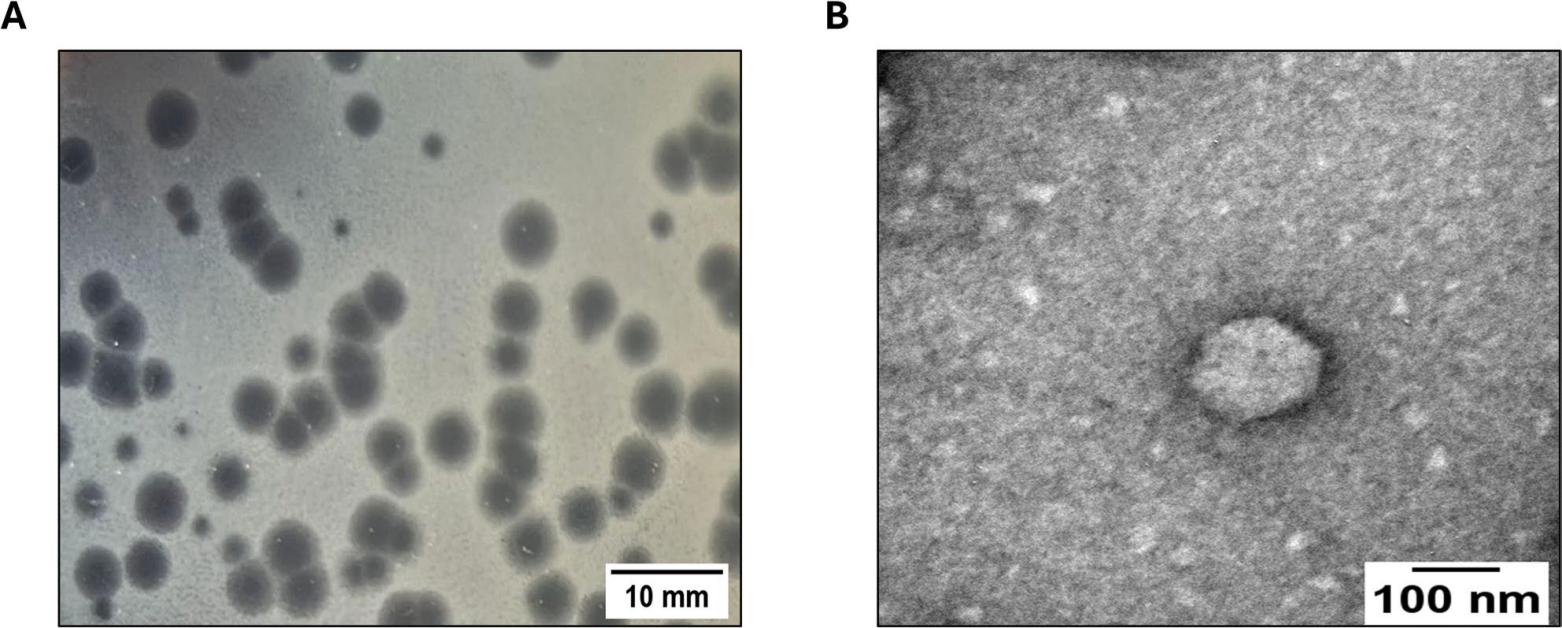
vB_PmiA_PM1 phage morphological characterization. **A **Phage plaques formed on the double-layer agar plates; **B **Transmission electron micrograph of phage particles after being negatively stained with 2% phospho-tungstic acid. The scale bar = 100 nm

A novel lytic phage, designated as vB_PmiA_PM1, was successfully isolated from sewage samples obtained from Zagazig University Hospitals (Zagazig, Sharkia Province, Egypt) using a clinical isolate of* P. mirabilis* (PM1) as a host. On DLA plate, vB_PmiA_PM1 phage formed clear plaques of 3.37 ± 0.11 mm in diameter surrounded by translucent halos (Fig. 2A). After purification and propagation, vB_PmiA_PM1 phage particles were visualized using TEM, which revealed virions with icosahedral heads and a very short tail (Fig. 2B). Based on these morphological characteristics and under the International Committee on Taxonomy of Viruses (ICTV) guidelines, vB_PmiA_PM1 phage was classified within the class *Caudoviricetes*.

### vB_PmiA_PM1 phage lytic spectra

vB_PmiA_PM1 phage lytic activity was investigated against 12 clinical *P. mirabilis* isolates in addition to a panel of other bacterial species. These included Gram-positive bacteria such as *Staph*ylococcus *aureus* (ATCC 6538) and a clinical isolate *Enterococcus faecalis* in addition to Gram-negative *Salmonella typhimurium* (ATCC 14028) and clinical isolates of *Pseudomonas aeruginosa*, *Serratia marcescens*, *Escherichia coli*, and *Klebsiella pneumoniae*. vB_PmiA_PM1 phage could only infect and lyse its main isolation host (PM1), whereas no visible change was detected with the other tested bacteria, indicating the absence of lytic activity.

### vB_PmiA_PM1 phage biological characterization

**Fig. 3:**
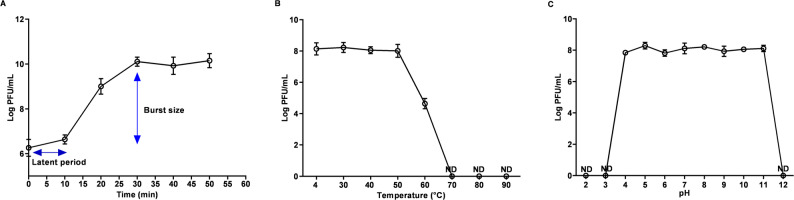
vB_PmiA_PM1 phage biological characterization. **A **One-step growth curve of vB_PmiA_PM1 phage. **B **vB_PmiA_PM1 phage tolerated temperatures up to 50 °C. **C **vB_PmiA_PM1 phage was stable over wide pH ranges (4–11); PFU, plaque-forming unit; ND, not detected

The one-step growth curve of vB_PmiA_PM1 phage revealed a short latency of approximately 10 min with a burst size of 211.6 virions/infected cell (Fig. 3A). Thermal stability test showed that it could withstand temperatures of up to 50 °C. However, a significant reduction in phage titer was recorded at 60 °C, with complete inactivation at higher temperatures (Fig. 3B). Additionally, the phage retained its activity over a broad pH range (4 to 11), while no phage activity was observed under extremely acidic (pH 2 and 3) and alkaline conditions (pH 12), as shown in Fig. 3C.

### Molecular characterization of vB_PmiA_PM1 phage and putative depolymerase identification

**Fig. 4:**
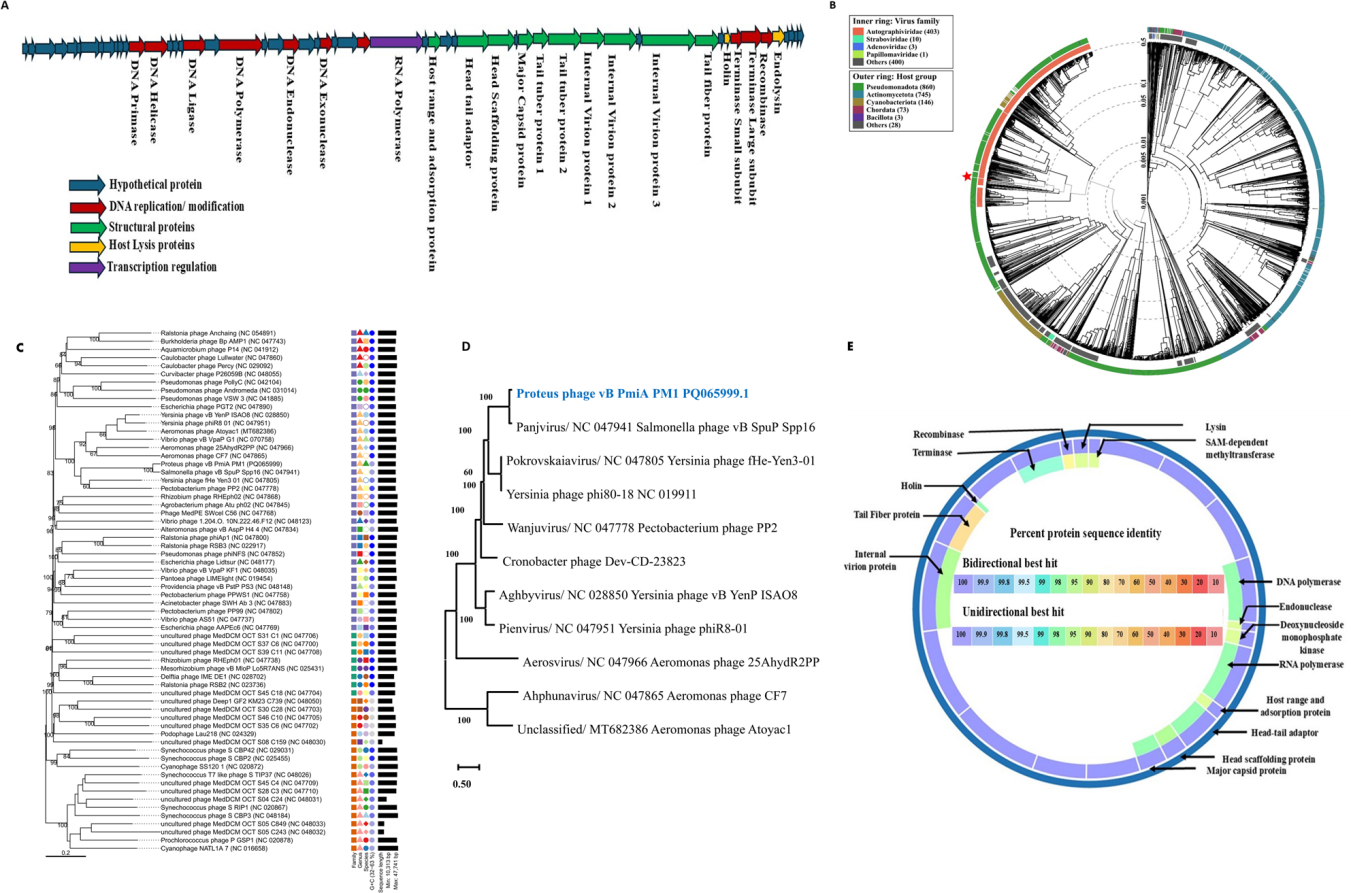
Whole genome annotation and bioinformatics analysis. **A **Genomic linear map of phage vB_PmiA_PM1. The coding sequences (CDS) were represented by different colors according to the category of the predicted function. **B **Circular proteomic tree based on genome-wide similarities of phage vB_PmiA_PM1 (marked with a red star), top matches on BLASTn, and closely related reference phage genomes. **C **VICTOR genome-based phylogenetic tree conducted by nucleotide pairwise comparison of phage vB_PmiA_PM1, other *Caudoviricetes* phages, and closely related phages according to genome BLASTn top hits. **D **Comparison of phage vB_PmiA_PM1, other references genome for different species of *Autographiviridae* viruses. The GTR + G model was used in MEGA11 with the lowest BIC scores (Bayesian Information Criterion). **E **Protein comparison between phage vB_PmiA_PM1 and the closely related genome *Salmonella* phage vB_SpuP_Spp16, showing percentage of variation in different genome parts

The complete genome sequencing of *P. mirabilis* phage vB_PmiA_PM1 was deposited in GenBank under accession number PQ065999. The genome is a linear double-stranded DNA of 42,188 bp including genes encoding hypothetical proteins, structural proteins, host lysis proteins, transcription and regulation proteins, and DNA replications, and modifications proteins (Fig. 4A and Supplementary Table S1). Direct BLASTN revealed close nucleotide relation with *Salmonella* phage vB_SpuP_Spp16 (NC_047941.1) based on 93% query coverage with 94.36% identity. Moreover, the protein of vB_PmiA_PM1 phage showed similarity to specific regions of the *P. mirabilis* reference genome including DNA polymerase (WP_368914334), endonuclease (WP_368914329.1), RNA polymerase (WP_368914324.1), tail fiber protein (WP_368914353.1) and terminase large subunit (WP_368914355.1) with identity percentages of 98.30, 91.86, 98.17, 73.13, and 98.53, respectively. Phage vB_PmiA_PM1 and the most closely related phages (NC_047941.1) were clustered among other *Autographiviridae* viruses whose bacterial hosts belong to class *Pseudomonadota* in VipTree analysis (Fig. 4B). The phylogenetic analysis based on the genome-to-genome distance calculator by VICTOR analysis generated 62 species clusters, 31 genus-level clusters, and three families. VICTOR clustered phage vB_PmiA_PM1 with other *Autographiviridae* viruses (Fig. 4C).

Analysis for safety using the PhageLeads tool did not detect any predicted temperate lifestyle genes or any antimicrobial resistance or virulence genes. The comparative genomic map of phage vB_PmiA_PM1 proteins and the closely related genome of *Salmonella* phage vB_SpuP_Spp16, represented by BV-BRC^3.46.3^ revealed that the most variable regions were in tail fiber protein (72.15%), recombinase (80.62%) followed by SAM-dependent methyltransferase (90.85%), lysin (92.86%), endonuclease (92.05%), deoxynucleoside monophosphate kinase (93.44%), and adsorption protein (93.43%) (Fig. 4D & E).

**Fig. 5:**
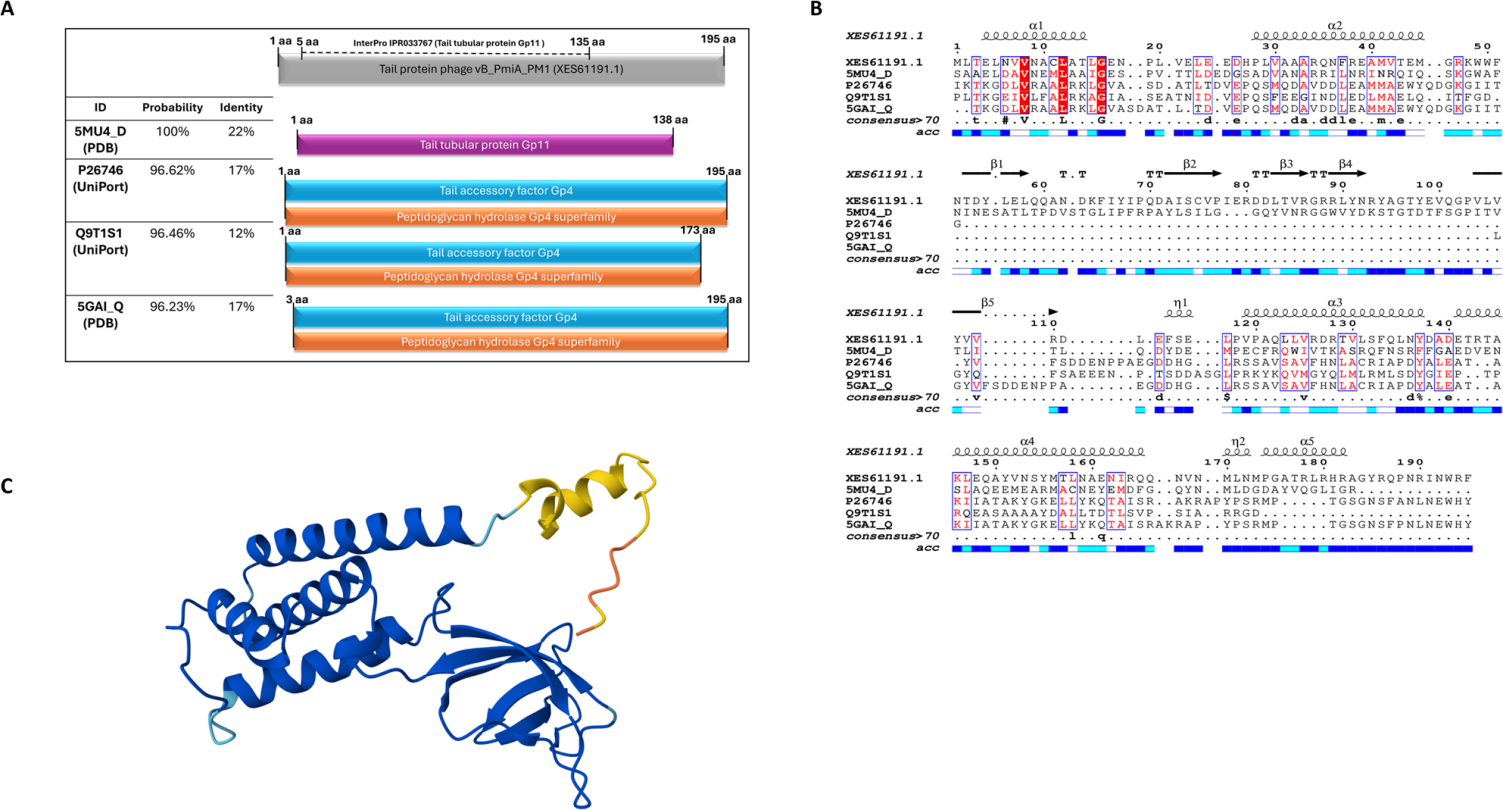
Putative depolymerase bioinformatic analysis. **A **Putative domains of phage tail protein (XES61191) predicted by HHpred. **B **The sequence alignment of phage tail protein with homologus protein. The figure was generated using ESPript. A schematic representation of the secondary structure elements is shown above the sequences. **C **The 3D structure model of phage tail protein (XES61191) generated by AlphaFold

ORF 43, ORF 44, and ORF 49 in vB_PmiA_PM1 phage genome are characterized as putative tail/ spike proteins and hypothesized to encode depolymerase enzymes that degrade bacterial capsular polysaccharide and biofilm extracellular polymeric matrix. Among the tail/spike proteins encoded by vB_PmiA_PM1 phage, ORF 43 was predicted to have a peptidoglycan hydrolase domain via Hhpred analysis (Fig. 5A). BLASTp analysis indicated that ORF 43 encoded protein (XES61191) shares high sequence homology with the tail protein of *Salmonella* phage vB_SpuP_Spp16 (YP_009799364) and a hypothetical protein from *P. mirabilis* phage (WP_368914317) exhibiting 100% and 99.74% identity with full sequence coverage, respectively. Notably, vB_PmiA_PM1 phage and *Salmonella* phage cluster within the same branch of the phage genome-based phylogenetic tree. InterProScan of tail protein (XES61191) revealed a conserved N-terminal residue (5-135 aa) belonging to tail tubular protein Gp11 (IPR033767), which acts as an adaptor protein. At the C-terminal, a conserved catalytic region within the alpha-helical domain in both the 5MU4_D and the ORF43 was observed via Phyre2, supporting functional homology. Pairwise sequence alignment and secondary structure analysis were visualized using ESPript (Fig. 5B). The 3D structure of the XES61191 protein was properly modelled using AlphaFold server (Fig. 5C), showing helical domains, ß strands, and loops. ExPasy-ProtParam predicted the physicochemical properties, the molecular weight of which was 22.8 kDa and the theoretical isoelectric point was 5.51. It has 195 amino acids, including 25 negatively charged and 22 positively charged. The Expasy-Protscale protein hydrophobicity analysis showed the maximum and minimum hydrophobicity values were 4.500 and − 4.500, respectively. TMHMM and SignaIP did not predict any transmembrane structural domains or signal peptide sequences.

### Phage stability and bacteriolytic activity in AUM

While a nutrient-rich medium (such as TSB) is widely used for studying the phage-host interactions in vitro, chemically defined urine ideally simulates the nutrient-limited and ion-rich conditions of the urinary tract, leading to substantial differences in biofilm formation, crystal precipitation, and phage activity. Accordingly, vB_PmiA_PM1 phage stability and bacterial viability were investigated in two AUM formulations over 24 h to simulate the urinary tract environment. The AUM formulation outlined by [30] was found to maintain phage viability and support bacterial growth, demonstrating no significant differences in either the phage titers or the bacterial counts over a 24-hour period (data not shown). Consequently, this formulation was selected for subsequent experiments. Moreover, the interaction between vB_PmiA_PM1 phage and *P. mirabilis* strain was assessed in AUM and TSB at different MOIs (10, 100, and 1000). Remarkably, TSB exhibited higher bacterial growth than AUM. Moreover, increasing the phage titer did not enhance vB_PmiA_PM1 phage lytic activity in either medium. Furthermore, a significant reduction in the growth rates of phage-treated bacteria was observed compared to the untreated controls.

**Fig. 6:**
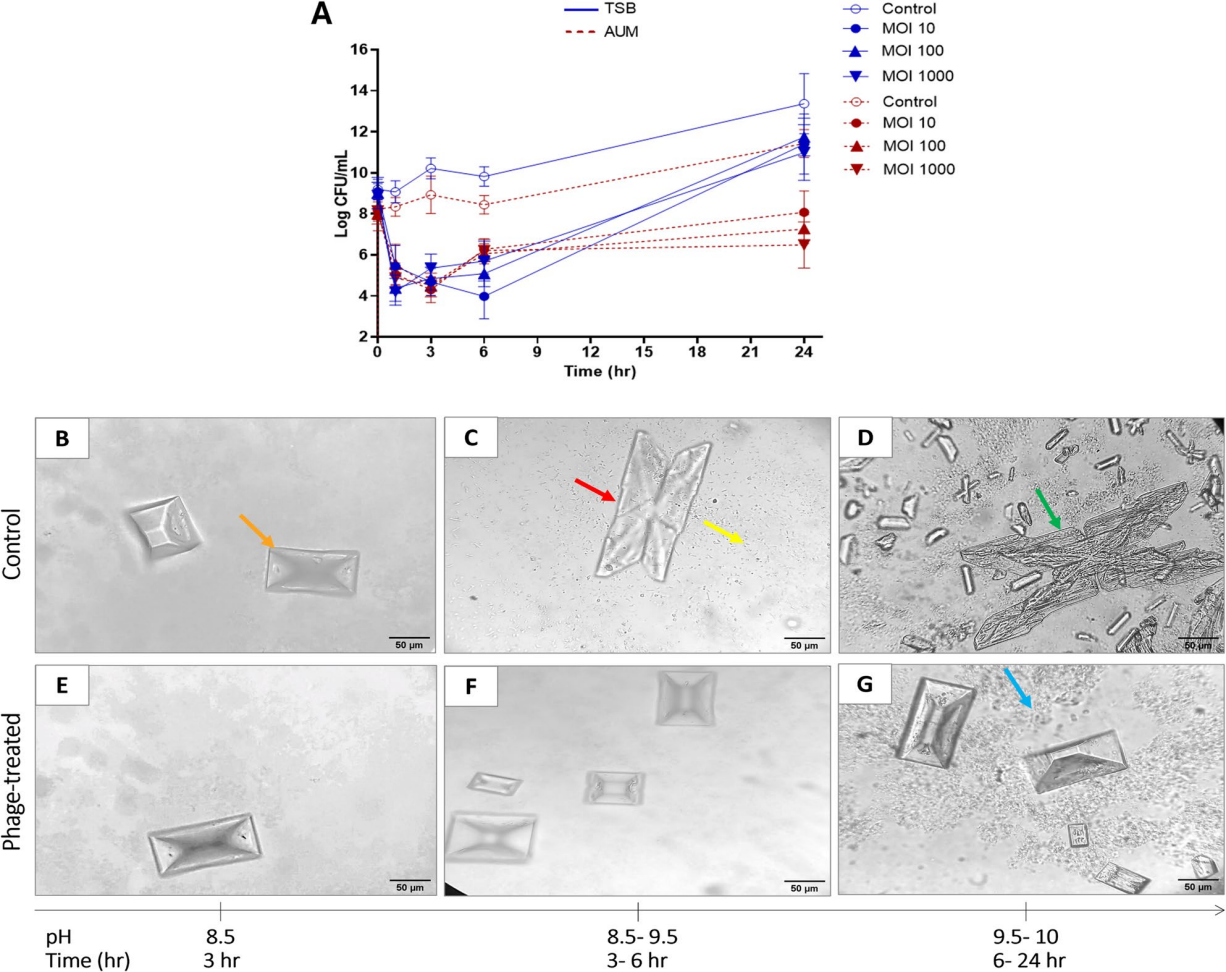
**(A)** The vB_PmiA_PM1 phage’s bacteriolytic activity against the host strain *P. mirabilis* in AUM and TSB over 24 h at different MOIs (10, 100, and 1000). Bacterial growth inhibition was assessed and expressed as CFU/mL; MOI, multiplicity of infection; CFU, colony-forming unit. pH variations and crystal formation caused by *P. mirabilis* in AUM over 24 h of incubation in the absence (panels **B**-**D**) and presence of vB_PmiA_PM1 phage at an MOI of 1000 (panels **E**-**G**). Apatite crystals (blue arrow); coffin-lid struvite crystals (orange arrow); twins (red arrow); dendritic struvite crystals (green arrow); bacteria (yellow arrow). The quality of images is enhanced using ImageJ to enable the identification of crystals and microorganisms. The scale bar = 50 nm

No discernible change in phage lytic activity was detected in TSB after 24 h. In AUM, bacteria treated with vB_PmiA_PM1 phage exhibited maximum bacteriolytic activity (approximately 4 log reduction) after 3 h of phage challenge at all MOIs tested, with equilibrium being reached after 6 h. Conversely, in TSB at 10 MOI, vB_PmiA_PM1 phage displayed bacteriolytic activity within 6 h, a result that was achieved after only 1 h with the higher MOIs (Fig. 6A).

### pH and crystal morphology

The impact of vB_PmiA_PM1 phage on host strain-induced pH variations and crystal formation was investigated. No significant difference was observed in pH levels between the control and phage-treated samples for either medium. Notably, rapid alkalization was observed in AUM due to urea hydrolysis by the urease enzyme, which subsequently induced crystallization.

In phage-untreated AUM, initial crystallization manifested as coffin-lid-shaped struvite crystals after almost 3 h at a pH of around 8.5. As time progressed and pH increased, the nature of the crystals remained unchanged, though twinning was observed. At the highest pH value (10), large dendritic struvite crystals appeared. Nevertheless, coffin-lid crystals were primarily observed in AUM treated with phage at all tested time intervals, with a relative increase in number. Additionally, apatite crystals were apparent after 24 h in both control and phage-treated AUM as microcrystalline aggregations (Fig. 6B and G). It is noteworthy that struvite crystals together with apatite constitute what are called the infectious stones, or struvite stones, where apatite makes up the minor component (up to 10%). Struvite stones can readily grow within a few weeks or months, forming a large stone that occupies the entire renal system. Patients with such infection-related stones are at high risk of losing the affected kidney if left untreated.

### Biofilm eradication capability of vB_PmiA_PM1 phage

**Fig. 7:**
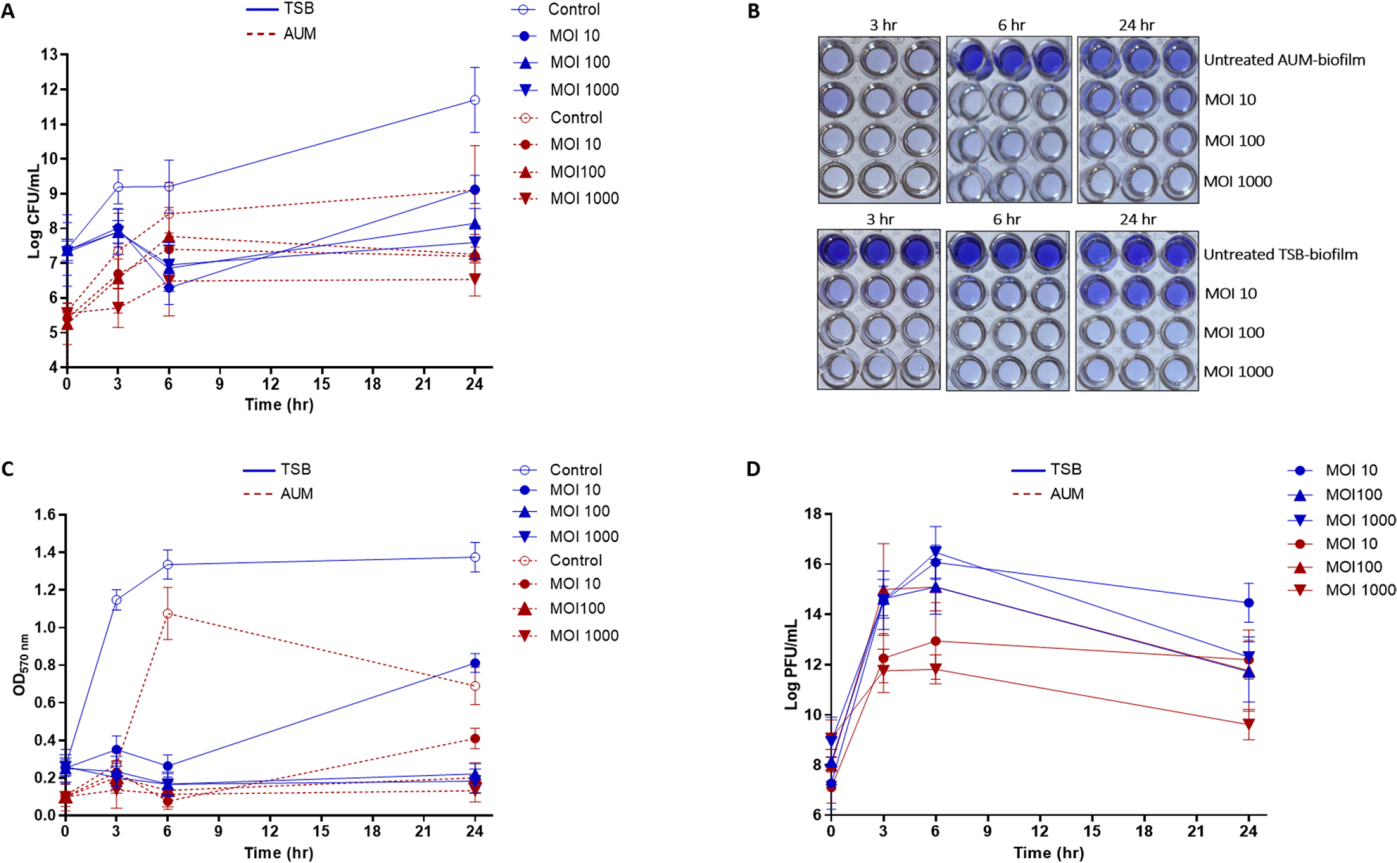
Mature biofilm eradication in AUM and TSB after challenge with vB_PmiA_PM1 phage over a 24-hour period at different MOIs (10, 100, and 1000). **A **The effect of vB_PmiA_PM1 phage on biofilm cell viability was quantified and expressed as CFU/mL. **B **Crystal violet staining of biofilm produced in 96-well plates, followed by **(C)** total biomass assessment at OD _570 nm_. **D **The changes in vB_PmiA_PM1 phage titer during treatment

The ability of vB_PmiA_PM1 phage to eliminate 24-hour-old biofilms was assessed in AUM and TSB with respect to CFU count and total biomass. Notably, TSB supported more abundant biofilm formation than AUM. Additionally, the reduction in bacterial CFU in both media was not significantly affected by increasing the phage titer. Interestingly, a gradual increase in bacterial CFU was observed in AUM during the initial 6 h, followed by a slight reduction (approximately 2.6 log) in the bacterial count for the highest MOI after 24 h. In contrast, TSB demonstrated maximum bacterial CFU reduction within 6 h of phage treatment; however, the bacterial regrowth was evident by the end of the experiment (Fig. 7A). Moreover, vB_PmiA_PM1 phage effectively reduced the biofilm biomass, with a more pronounced effect observed after 6 h in both media. The percentage reductions were approximately 92.9%, 87.6%, and 89.5% in AUM, and about 80.3%, 87.5%, and 87.6% in TSB at MOIs of 10, 100, and 1000, respectively. Interestingly, AUM-biofilm growth was compromised with time. After 24 h, bacterial regrowth was observed with the lowest MOI in both media (Fig. 7B and C).

The use of DLA technique for phage enumeration showed that vB_PmiA_PM1 titers increased by several orders of magnitude in both AUM and TSB media within 6 h of phage infection, after which a gradual decrease in phage titer was noticed (Fig. 7D). Collectively, these results revealed the disparity between outcomes observed in TSB versus AUM, which could help elucidate the failure of phage therapy in biofilm-associated infections.

### vB_PmiA_PM1 phage efficacy on SFC biofilm structure and swarming migration

**Fig. 8:**
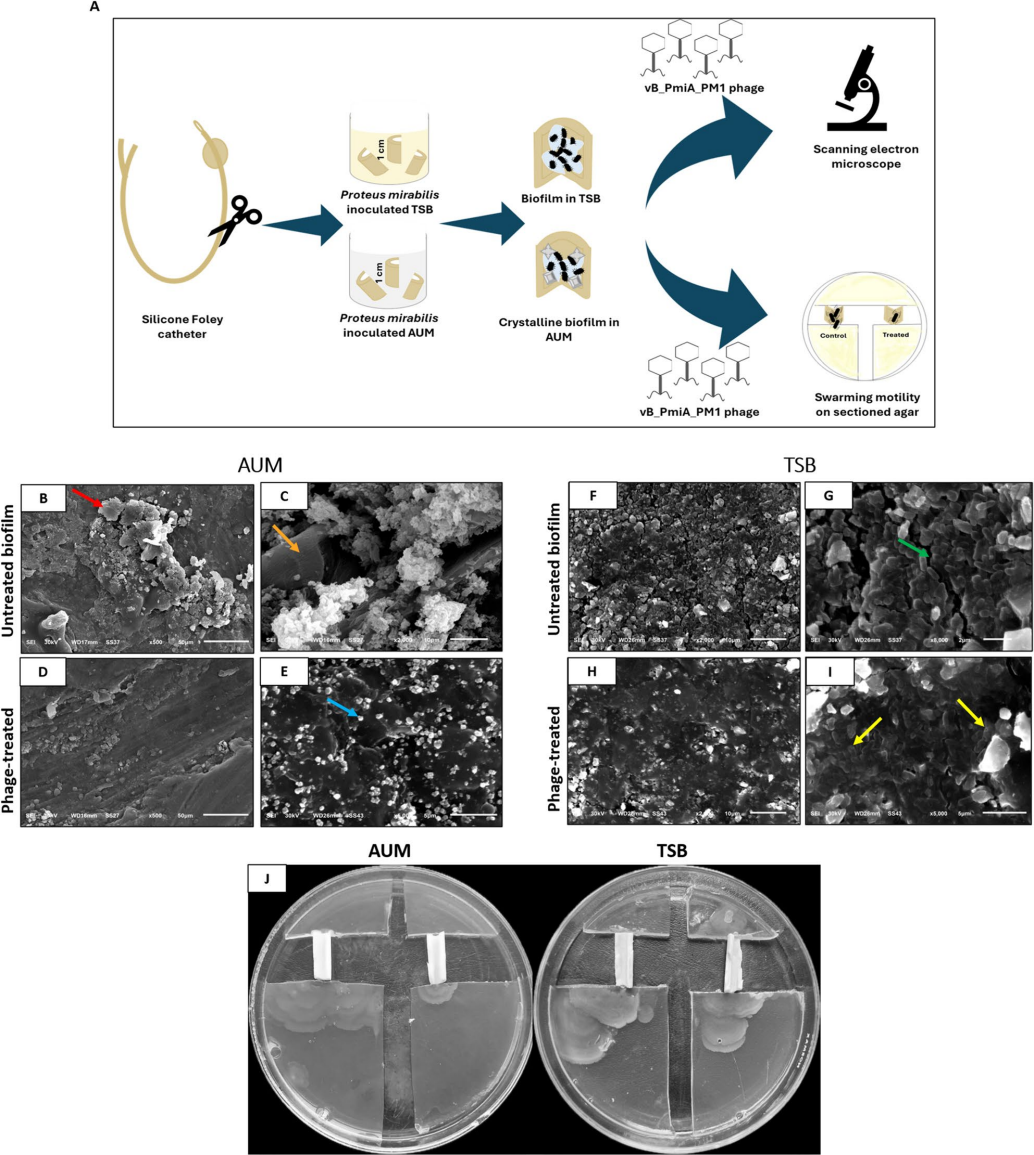
(**A**) Schematic overview of the experimental methodology. Panels (**B** and **C**) Untreated AUM-biofilm showing powdery appearance of apatite crystals, along with hexagonal-shaped (red arrow) and massive, dense, and porous struvite crystals (orange arrow), scale bars = 50 μm (**B**) and 10 μm (**C**); panels (**D** and **E**) vB_PmiA_PM1 phage-treated biofilm in AUM (MOI = 1000) with an obvious decrease in the biofilm matrix; however, encrustation of apatite crystals was observed (blue arrow), scale bars = 50 μm (**D**) and 5 μm (**E**); panels (**F** and **G**) Untreated TSB-biofilm where bacterial cells are covered with an extensive biofilm matrix with many channels embedded in the biofilm (green arrow), scale bars = 10 μm (**F**) and 2 μm (**G**); panels (**H** and **I**) vB_PmiA_PM1 phage-treated biofilm in TSB (MOI = 1000) showing reduced biofilm matrix, altered cell morphology, and pore formation (yellow arrows), scale bars = 10 μm (**H**) and 5 μm (**I**). **(J)**Migration of *P. mirabilis* on vB_PmiA_PM1 phage-treated SFC sections in AUM and TSB. In each plate, the untreated control is positioned on the left side, while the treated catheter section is on the right side. The quality of images is improved using ImageJ to improve visualization

SEM analysis of vB_PmiA_PM1 phage activity against SFC biofilm revealed substantial differences across treatment conditions. *P. mirabilis* exhibited a less dense biofilm structure in AUM, almost devoid of nutrient channels, and contained crystalline deposits. The crystals had an amorphous powdery appearance, predominantly attributed to apatite crystals, along with noticeable, large, dense, and hexagonal-shaped struvite crystals. Interestingly, the porous structure of struvites was also apparent (Fig. 8B and C). However, in TSB, *P. mirabilis* produced a robust biofilm architecture characterized by extensive aggregation of bacterial cells embedded in extracellular matrix, interspersed with numerous channels and crystalline-like depositions (Fig. 8F and G).

Treatment with vB_PmiA_PM1 phage in AUM led to a discernible decrease in biofilm matrix, though encrustation of apatite crystals was still observed (Fig. 8D and E). A considerable reduction in biofilm matrix was also observed in TSB following phage treatment, along with obvious morphological alterations in bacterial cells, including distorted cell morphology and pore formation in some cells, indicative of phage-mediated lysis (Fig. 8H and I). Phage activity against *P. mirabilis* swarming over SFC sections was also assessed in both media. Interestingly, in AUM, the vB_PmiA_PM1 phage-treated catheter exhibited slower bacterial migration across the catheter bridge compared to the untreated control. In contrast, swarming was clearly visible in TSB despite phage treatment (Fig. 8J).

## Discussion

Catheter-associated urinary tract infection (CAUTI) is the most prevalent hospital-acquired infection and represents a global health concern due to its long-term morbidity and recurrence [35]. One of the chief causative agents of CAUTIs is the uropathogen *P. mirabilis*, which is characterized by its capability to cause catheter encrustation and blockage. Notably, *P. mirabilis* was previously reported to have the highest propensity among Gram-negative bacteria to attach to the catheter surface and adapt to the catheterized urinary tract. Proteus-mediated CAUTIs demonstrate unusual characteristic sequelae, including crystalline biofilm formation on catheter surface and urinary epithelial cells, as well as the development of urinary stones [36]. These crystalline biofilms and stones serve as a protective shield for bacterial cells against immune cells and antibiotics, in addition to acting as a nidus for reinfection after catheter removal. Phage therapy in CAUTIs has shown promising efficacy in the laboratory [37], but clinical trials have not reflected these findings. This sharp divergence between phage assessment outcomes could be attributed to the complex phage-host interactions encountered in the intricate environment of the catheterized urinary tract [38]. Thus, the current study aimed to bridge the gap between the in vitro and in vivo applications of phage therapy in CAUTIs.

In the current study, a new lytic phage, designated vB_PmiA_PM1, was isolated from a sewage pump station targeting MDR *P. mirabilis* associated with urinary tract infection. The morphological examination of the phage by TEM revealed an icosahedral head with a very short tail, classifying it within the class *Caudoviricetes.* As in previous studies [22, 39], the vB_PmiA_PM1 phage demonstrated high host specificity, exhibiting efficient lytic activity against its host strain with no lytic activity against other *P. mirabilis* clinical isolates or other bacterial species tested. However, the limited number of *P. mirabilis* clinical isolates included in the current study could explain these findings. The biological characterization of vB_PmiA_PM1 phage revealed a short latency period and substantial burst size along with thermal and acid-base tolerance. These features render vB_PmiA_PM1 phage a promising candidate for biocontrol approaches.

Molecular analysis of putative novel phages is crucial for assessing their safety by determining potential risks within the genome. These potential risks include resistance and virulent genes or lysogeny encoding genes [40]. Although bioinformatic analysis can minimize the risk of using a novel phage and the limitless trial for safety testing, the presence of many genes with unknown functions (hypothetical proteins) still limits their public use. The whole genome sequence of vB_PmiA_PM1 phage indicated no known genes encoding virulence factors, antimicrobial resistance, or predicted temperate lifestyle. Hence, this phage can potentially be used for therapeutic purposes [41]. The significance of vB_PmiA_PM1 phage was supported by the detection of holins which are small membrane proteins responsible for creating holes in bacteria`s cytoplasmic membranes, allowing the endolysins to access and disrupt peptidoglycan to complete the lytic cycle of most bacteriophages [42]. The endolysin-holin-endopeptidase genes usually correlate with the lysis function of tailed phages during the terminal stage of their lytic cycle [43]. Despite the vB_PmiA_PM1 phage’s close relationship with *Salmonella* phage vB_SpuP_Spp16, it did not show any activity against the tested *Salmonella* isolates. Although some proteins were similar to the *Proteus* reference genome, they did not match the same classification. These facts may indicate a high incidence of horizontal gene transfer and mutation [44].

Considering the biofilm nature and the role of bacterial exopolysaccharides in biofilm formation, phages equipped with depolymerase enzymes are presumed to be effective against bacterial biofilm [45]. Phage vB_PmiA_PM1 produced clear plaques surrounded by a translucent halo zone. This halo zone demonstrates the degradation of bacterial surface polysaccharides by the phage depolymerase enzyme that may be present in tail tubular protein, fiber or, spikes [46]. Structural analysis of the tail protein (XES61191) identified a peptidoglycan hydrolase domain, displaying structural homology to tail tubular protein A of *Klebsiella pneumoniae* bacteriophage KP32 (PDB ID: 5MU4_D), previously identified as α-1,4-glucosidase [47]. The conserved catalytic motif (Asp-X-Asp) reported in 5MU4_D, responsible for glycosyl hydrolysis, is also present in the protein encoded by ORF 43, supporting its predicted enzymatic activity [48]. Furthermore, HHpred analysis showed that both proteins encoded by ORF 43 and tail adaptor protein Gp4 share a peptidoglycan hydrolase domain [47, 49]. The lack of transmembrane domain or signal peptide prediction further supports its role as a tail structural protein that exerts its enzymatic activity upon host contact. Protein physiochemical properties prediction demonstrated a molecular weight of 22.8 kDa and a theoretical isoelectric point of 5.5. This slightly acidic nature may affect protein activity depending on the pH of the surrounding medium. The putative depolymerase activity of vB_PmiA_PM1 phage-encoded protein underscores its potential utility as an anti-proteus therapy against antibiotic-resistant species and for biofilm degradation [50].

*P. mirabilis* has considerable metabolic adaptability and versatility, enabling it to endure nutritional limitations in the urinary tract and establish pathogenesis [11]. The interaction between phage vB_PmiA_PM1 and *P. mirabilis* host bacterium was assessed under conditions that closely mimic the urinary tract and was compared to laboratory-rich media. Given the high complexity and individual variability in the composition of human urine, AUM were employed in the current study. To optimize the experimental conditions, the stability of vB_PmiA_PM1 phage and host viability was evaluated in two AUM formulations, as described by [30] and Sarigul *et al*. [31]. Notably, the AUM described by [30] fulfilled the requirements for *P. mirabilis* growth and vB_PmiA_PM1 phage stability and was considered the medium of choice for the current study. Similarly, previous studies reported phage titer stability in AUM without any fluctuations [51, 52].

Regarding the bacterial host, growth was notably more pronounced in TSB compared to AUM, likely due to the limited nutrition in AUM. Alternatively, abrupt bacteriolytic activity of phage vB_PmiA_PM1 was observed within 3 h in AUM across all tested MOIs. However, in TSB, the lowest MOI (10) exhibited delayed bacteriolytic activity. An equilibrium was reached in AUM after 6 h, which could be attributed to the counterbalance between phage bacteriolytic activity and bacterial regrowth or the emergence of phage-resistant mutants. These findings aligned with [38], who attributed the growth attenuation of phage-resistant mutants in AUM to nutrient deprivation, which could be restored through nutrient supplementation. Conversely, bacterial host rebound in TSB exceeded phage bacteriolytic activity. Previous reports on phage-host coevolution suggest that the existence of host cells protected from phage infection could be due to fluctuations in the expression of phage receptors [53] or reduced killing of stationary-phase cells [54]. The variation in phage-host dynamics in AUM and laboratory media could justify the misleading outcomes in the assessment of phage therapy using laboratory media as observed in cases of personalized phage therapy failure.

A major complication of CAUTIs is the encrustation of the catheter surface, which can ultimately lead to obstruction of the catheter lumen and urinary retention. Through urine alkalinization, the uropathogen *P. mirabilis* induces the precipitation of urine minerals, resulting in extensive crystallization and catheter blockage within 24 h [55]. These sequelae are driven primarily via the urease enzyme, which breaks down urea and releases ammonia [56]. In the current study, the pH of the media was monitored during phage-host interaction. Interestingly, pH increased drastically to become alkaline (pH 10) in the untreated and phage-treated AUM. This pH shift in phage-treated AUM is likely to influence the capsular polysaccharide hydrolytic activity of the putative depolymerase enzyme via altering the ionization state of catalytic residues such as aspartate, which in turn hinders phage-host attachment. Notable variations in struvite crystal morphology were evident. In phage-treated AUM, coffin-lid crystals were predominantly present, showing a tendency to increase in number and form aggregates rather than large crystals caused by urease-derived alkalization. This basic coffin-lid crystal morphology is discrete, less adherent, and more easily dispersed and could be beneficially removed by urine flow. From a clinical perspective, these findings suggest that this approach may help maintain catheter patency and reduce the incidence of catheter-associated blockage. In contrast, untreated AUM exhibited distinct crystal morphologies correlated with pH increase over time. Such large crystals are entrapped within the catheter lumen, promoting nucleated crystallization and eventual catheter blockage. A plausible explanation for differences in crystal morphology and intensity is that crystallization does not depend solely on urease enzyme activity. The chemical nature and structure of free bacterial endotoxin released from dead cells, in this case through phage-mediated cell lysis, also play a role in the crystallization of urine components [57].

Microbial biofilms exhibit a substantial compositional diversity tailored to their specific environmental niches. In the context of the crystalline biofilm involved in CAUTIs, *P. mirabilis* was allowed to form biofilm in either AUM or TSB. Notably, bacterial biofilm formation was more pronounced in TSB. This phenomenon may be attributed to nutrient deprivation or limited bacterial growth caused by the rise in pH over time, as previously reported [20]. Furthermore, the nutrient scarcity in AUM likely triggered biofilm cell detachment, as evidenced by the untreated biofilm’s gradual increase followed by a significant reduction over the tested time intervals. Detached cells can disseminate to new sites, facilitating the colonization of distant tissues and indwelling devices [20]. Phage vB_PmiA_PM1 demonstrated significant biofilm-eradicating activity in TSB, as evidenced by a reduction in both CFU and total biomass, with a preference for the higher MOI. On the other hand, the phage’s bacteriolytic activity was completely compromised in AUM, despite a noticeable reduction in total biomass. The altered phage bacteriolytic activity in AUM could be attributed to the accumulation of apatite and struvite crystalline deposits that shield the embedded cells, creating a physical barrier, which hinders phage penetration and adsorption. Moreover, the physiological state of biofilm-embedded cells can also reduce phage activity, because cells in the logarithmic phase of growth are more efficiently lysed by phages than those in the quiescent metabolic state within the biofilm [58]. Another explanation is that the capsular polysaccharide-degrading activity of depolymerase may be diminished in alkaline conditions, resulting in limited contact and attachment between bacterial receptor and phage tail. In line, phage-derived depolymerase DPO42 was reported to completely lose its activity at pH values above 8 [59].

The continued development of biofilms and deposition of crystalline materials often leads to obstruction of urine flow through the catheter lumen. Urinary incontinence may develop due to catheter leakage, and infected urine reflux can lead to pyelonephritis, endotoxic shock, and septicemia. Additionally, these encrustations can shield the embedded biofilm cells, thereby hindering phage adsorption [58]. For further validation, a Foley catheter, the most commonly used indwelling catheter, was utilized as an abiotic surface for biofilm formation, and biofilm architecture was examined via SEM. *P. mirabilis* biofilm encrusted the Foley catheter in AUM with a characteristic powdery appearance, primarily composed of apatite crystals associated with massive struvite crystals. However, biofilm in TSB was denser, with bacterial cells extensively covered by biofilm matrix and interspersed with crystal-like deposits. These findings were aligned with [20], who reported different biofilm morphotypes in laboratory media and AUM. Moreover, phage treatment in AUM led to a noticeable reduction in the biofilm matrix, although apatite encrustations remained observable. A possible explanation for these findings is that limited nutrients fasten bacterial detachment through EPS degradation, which is responsible for binding mineral deposits that encrust the catheter surface. This disruption likely caused the release of large, dense struvite crystals into the surrounding media, while smaller apatite crystals remained adherent to the catheter surface.

Cell-to-cell signaling in biofilm and flagellar contact with a solid surface are key factors mediating bacterial swarming motility. *P. mirabilis* swarmer cells successfully migrated over urinary catheters compared to other uropathogens, ultimately reaching the kidney and contributing to complicated CAUTIs [60]. In the current study, phage vB_PmiA_PM1 treatment of AUM-biofilm slowed the bacterial migration rate. Notably, slower swarmer cells prolong exposure to host immune responses and bactericidal agents, potentially leading to fewer territory outcomes. In contrast, bacterial migration in TSB was more pronounced even after phage treatment. In this context [61], demonstrated that quorum-sensing signals induced phage defense mechanisms with the evolution of a sub-population of phage-tolerant cells. However, this phage-host coevolution occurs at the expense of temporarily altering the bacterial primary function such as motility. Despite the valuable insights provided in the present study, we are aware of certain limitations. A definitive conclusion about phage efficacy requires testing against a larger number of *P. mirabilis* isolates and other bacterial species. However, phage host specificity could be advantageous in phage therapy application, potentially limiting the development of community-wide resistance. Phage infection assay was evaluated via a spot assay rather than efficiency of plating (EOP). Despite the spot assay is recommended for preliminary screening, EOP analysis would provide a more indicative assessment of phage infectivity. Second, data on depolymerase activity are based only on observations of plaque morphology and bioinformatic analysis; despite these being strong evidence, further investigation involving recombinant production of this protein with characterization of its biochemical properties, structural features, and therapeutic effectiveness is warranted. In conclusion, to the best of our knowledge, the current study is the first to comprehensively evaluate phage bacteriolytic and biofilm-eradicating activity in both AUM and laboratory-rich media, highlighting the pivotal influence of environmental conditions on phage therapy outcomes in CAUTIs. Conventional in vitro assessment of phage therapy using laboratory media underestimates the crystalline biofilm and catheter encrustation, which are principal factors in the CAUTI pathogenesis. Future studies should prioritize evaluating phage therapy under conditions that closely mimic the intricate environment of the catheterized urinary tract to adequately anticipate the clinical outcome of phage therapy in CAUTIs.

## Supplementary Information

Supplementary Material 1.

## Data Availability

The complete genome sequence of *Proteus mirabilis* phage vB_PmiA_PM1 has been submitted to GenBank under accession number PQ065999.

## Abbreviations

CAUTI: Catheter-associated urinary tract infection
AUM: Artificial urine media
TSB: Tyrpticase soya broth
TSA: Tyrpticase soya agar
PFU/mL: Plaque-forming units per milliliter
TEM: Transmission electron microscope
MOI: Multiplicity of infection
BV-BRC: Bacterial and viral bioinformatics resource center
PBS: Phosphate-buffered saline
SFC: Silicone Foley catheter
SEM: Scanning electron microscopy
ICTV: International Committee on Taxonomy of Viruses
EOP: Efficiency of plating
