# Supplementary material for: Evaluating Proteus mirabilis phage vB_PmiA_PM1 efficacy against catheter-associated urinary tract infections in artificial urine and laboratory media
Source: BMC Microbiol. 2026 Mar 6;26:390. doi: 10.1186/s12866-026-04843-w (PMC13097943; doi:10.1186/s12866-026-04843-w)
Supplement: Supplementary file 1 — Supplementary Material 1. [file 12866_2026_4843_MOESM1_ESM.docx]

**Supplementary Table S1: Detailed genome annotation of phage vB_PmiA_PM1**

| **ORF** | **Product** | **Start** | **End** | **Strand** | **Protein ID** | **Length** | **Amino acid length** |
| --- | --- | --- | --- | --- | --- | --- | --- |
| ORF1 | Hypothetical protein | 314 | 460 | + | XES61149.1 | 147 | 48 |
| ORF2 | Hypothetical protein | 527 | 604 | + | XES61150.1 | 78 | 25 |
| ORF3 | Hypothetical protein | 1074 | 1913 | + | XES61151.1 | 840 | 279 |
| ORF4 | SAM-dependent methyltransferase | 1960 | 2388 | + | XES61152.1 | 429 | 142 |
| ORF5 | Hypothetical protein | 2660 | 3001 | + | XES61153.1 | 342 | 113 |
| ORF6 | Hypothetical protein | 2925 | 3074 | + | XES61154.1 | 150 | 49 |
| ORF7 | Hypothetical protein | 3585 | 3737 | + | XES61155.1 | 153 | 50 |
| ORF8 | Hypothetical protein | 3748 | 4260 | + | XES61156.1 | 513 | 170 |
| ORF9 | Hypothetical protein | 4270 | 4476 | + | XES61157.1 | 207 | 68 |
| ORF10 | Hypothetical protein | 4488 | 5030 | + | XES61158.1 | 543 | 180 |
| ORF11 | Hypothetical protein | 5031 | 5276 | + | XES61159.1 | 246 | 81 |
| ORF12 | Hypothetical protein | 5158 | 5289 | + | XES61160.1 | 132 | 43 |
| ORF13 | Hypothetical protein | 5286 | 5828 | + | XES61161.1 | 543 | 180 |
| ORF14 | Hypothetical protein | 5828 | 6130 | + | XES61162.1 | 303 | 100 |
| ORF15 | Hypothetical protein | 6123 | 6452 | + | XES61163.1 | 330 | 109 |
| ORF16 | Hypothetical protein | 6449 | 6913 | + | XES61164.1 | 465 | 154 |
| ORF17 | DNA primase | 6906 | 7550 | + | XES61165.1 | 645 | 214 |
| ORF18 | DNA helicase | 7574 | 8641 | + | XES61166.1 | 1068 | 355 |
| ORF19 | Hypothetical protein | 8644 | 8817 | + | XES61167.1 | 174 | 57 |
| ORF20 | Hypothetical protein | 8762 | 9076 | + | XES61168.1 | 315 | 104 |
| ORF21 | Hypothetical protein | 9033 | 9317 | + | XES61169.1 | 285 | 94 |
| ORF22 | DNA ligase | 9314 | 10261 | + | XES61170.1 | 948 | 315 |
| ORF23 | hypothetical protein | 10265 | 10798 | + | XES61171.1 | 534 | 177 |
| ORF24 | DNA polymerase | 10798 | 13269 | + | XES61172.1 | 2472 | 823 |
| ORF25 | hypothetical protein | 13269 | 13415 | + | XES61173.1 | 147 | 48 |
| ORF26 | hypothetical protein | 13458 | 14258 | + | XES61174.1 | 801 | 266 |
| ORF27 | DNA exonuclease | 14267 | 15229 | + | XES61175.1 | 963 | 320 |
| ORF28 | hypothetical protein | 15233 | 15616 | + | XES61176.1 | 384 | 127 |
| ORF29 | Hypothetical protein | 15616 | 15834 | + | XES61177.1 | 219 | 72 |
| ORF30 | Endonuclease | 16014 | 16289 | + | XES61178.1 | 276 | 91 |
| ORF31 | Hypothetical protein | 16282 | 16515 | + | XES61179.1 | 234 | 77 |
| ORF32 | Hypothetical protein | 16508 | 17512 | + | XES61180.1 | 1005 | 334 |
| ORF33 | Deoxynucleoside monophosphate kinase | 17512 | 18063 | + | XES61181.1 | 552 | 183 |
| ORF34 | RNA polymerase | 18072 | 20531 | + | XES61182.1 | 2460 | 819 |
| ORF35 | Hypothetical protein | 20691 | 20864 | + | XES61183.1 | 174 | 57 |
| ORF36 | Host range and adsorption protein | 20864 | 21277 | + | XES61184.1 | 414 | 137 |
| ORF37 | Hypothetical protein | 21277 | 21681 | + | XES61185.1 | 405 | 134 |
| ORF38 | Hypothetical protein | 21681 | 21851 | + | XES61186.1 | 171 | 56 |
| ORF39 | Head-tail adaptor | 21848 | 23362 | + | XES61187.1 | 1515 | 504 |
| ORF40 | Head scaffolding protein | 23364 | 24173 | + | XES61188.1 | 810 | 269 |
| ORF41 | Hypothetical protein | 24251 | 24352 | + | XES61189.1 | 102 | 33 |
| ORF42 | Major capsid protein | 24370 | 25278 | + | XES61190.1 | 909 | 302 |
| ORF43 | Tail protein | 25362 | 25949 | + | XES61191.1 | 588 | 195 |
| ORF44 | Tail protein | 25951 | 28557 | + | XES61192.1 | 2607 | 868 |
| ORF45 | Internal virion protein | 28560 | 29318 | + | XES61193.1 | 759 | 252 |
| ORF46 | Internal virion protein | 29327 | 31648 | + | XES61194.1 | 2322 | 773 |
| ORF47 | Hypothetical protein | 31651 | 31755 | + | XES61195.1 | 105 | 34 |
| ORF48 | Internal virion protein with endolysin domain | 31758 | 35168 | + | XES61196.1 | 3411 | 1136 |
| ORF49 | Tail fiber protein | 35237 | 37024 | + | XES61197.1 | 1788 | 595 |
| ORF50 | Hypothetical protein | 36997 | 37146 | + | XES61198.1 | 150 | 49 |
| ORF51 | Holin | 37170 | 37385 | + | XES61199.1 | 216 | 71 |
| ORF52 | Terminase small subunit | 37354 | 37740 | + | XES61200.1 | 387 | 128 |
| ORF53 | Terminase large subunit | 37740 | 39653 | + | XES61201.1 | 1914 | 637 |
| ORF54 | Recombinase | 39653 | 40135 | + | XES61202.1 | 483 | 160 |
| ORF55 | Endolysin | 40171 | 40719 | + | XES61203.1 | 549 | 182 |
| ORF56 | Hypothetical protein | 40716 | 41042 | + | XES61204.1 | 327 | 108 |
| ORF57 | Hypothetical protein | 40936 | 41187 | + | XES61205.1 | 252 | 83 |
| ORF58 | Hypothetical protein | 41184 | 41342 | + | XES61206.1 | 159 | 52 |
| ORF59 | Hypothetical protein | 41832 | 42065 | + | XES61207.1 | 234 | 77 |
